# The Predictive Role of Bladder Ultrasound in Children with Nocturnal Enuresis: A Comprehensive Overview

**DOI:** 10.3390/children12040520

**Published:** 2025-04-17

**Authors:** Ignazio Cammisa, Pietro Ferrara

**Affiliations:** 1 Department of Life Sciences and Public Health, Fondazione Policlinico Universitario A. Gemelli IRCCS, 00168 Rome, Italy; 2 Department of Medicine and Surgery, Università Campus Bio-Medico, 00128 Rome, Italy; p.ferrara@policlinicocampus.it; 3 Operative Research Unit of Pediatrics, Fondazione Policlinico Universitario Campus Bio-Medico, 00128 Rome, Italy

**Keywords:** enuresis, children, bladder features, pediatric, ultrasound, therapy response

## Abstract

**Background/Objectives**: Nocturnal enuresis (NE) is a common condition in children, affecting more than 10% of 6-year-olds and typically characterized by involuntary bedwetting during sleep. Although the exact causes remain unclear, ultrasound has emerged as a valuable tool for assessing bladder function and predicting therapy response and relapse, with several studies linking urological abnormalities like reduced bladder capacity and increased wall thickness to treatment outcomes. **Methods**: We performed a literature review utilizing five distinct search queries on PubMed with the following keywords: “enuresis & ultrasound”, “enuresis & bladder ultrasound”, “enuresis & bladder wall”, “enuresis & bladder capacity”, and “enuresis & bladder thickness”. We reviewed each article that met the eligibility criteria, and after a thorough selection, we included 17 studies. **Results**: Bladder ultrasound is a valuable tool for assessing bladder function and structure in children with NE, providing crucial insights into bladder capacity, wall thickness, and the bladder wall thickness index (BVWI). These ultrasound measurements correlate with the severity of NE and treatment success, aiding in personalized therapy, particularly for children with smaller bladder capacities and thicker bladder walls. Although studies have explored the link between ultrasound findings and treatment response, further research is needed to standardize methods and confirm these connections. **Conclusions**: The review highlights the predictive role of ultrasound in enuresis management, focusing on the response rate and choice of therapy. Future studies should investigate how bladder imaging can predict both short-term and long-term treatment outcomes, with current evidence supporting its use in customizing NE treatment for improved results.

## 1. Introduction

According to the International Children’s Continence Society (ICCS), nocturnal enuresis (NE) is defined as involuntary intermittent bedwetting during sleep in children, typically those aged 5 years or older, with a higher incidence in males [1,2,3]. The prevalence of NE varies; it is observed in more than 10% of 6-year-olds, approximately 5% of 10-year-olds, and 0.5–1% of adolescents and young adults [1,2,3]. NE is classified into primary and secondary forms. Primary NE, the more common variant, refers to cases where the child has never experienced a period of continuous nighttime dryness lasting longer than 6 months. Secondary NE, on the other hand, occurs when involuntary urination recurs after at least a 6-month period of nighttime dryness [3,4,5]. Another important clinical classification of NE is into monosymptomatic (MNE) and non-monosymptomatic (NMNE) types. The term NMNE refers to children who, in addition to nighttime bedwetting, also experience daytime lower urinary tract symptoms (LUTS), such as urgency, daytime incontinence, difficulty voiding, and changes in the frequency of daytime urination [3,4,5]. The exact mechanisms of NE remain unclear, but various factors contribute to its onset and persistence, including delayed development, genetic factors, increased nighttime urine production, disrupted sleep, reduced bladder capacity, and overactive bladder muscles [6,7,8,9,10]. A key factor in primary NE appears to be delayed maturation of the central nervous system, supported by neurophysiological data [11]. Additionally, comorbidities are present in 20–30% of children with NE, which is 2 to 4 times more common than in control populations, and they may influence treatment outcomes and relapse rates [7,12,13,14]. Various pharmacological and non-pharmacological strategies have been suggested for managing NE, with the choice of treatment depending on factors such as the specific type of NE, the severity of the issue, the child’s willingness to participate, and the level of parental compliance [3,4,5]. First-line treatments typically include medications (such as desmopressin for MNE and anticholinergics for NMNE) along with behavioral interventions [3,4,5]. Moreover, the response to therapy and the risk of relapse appear to be closely associated with the morphological and structural characteristics of the bladder, as revealed by urodynamic investigations (UDS) and ultrasound [15,16,17]. Several studies have demonstrated that enuretic children often exhibit urological abnormalities, such as reduced bladder capacity, bladder hypocompliance, detrusor overactivity, and increased bladder wall thickness [18,19,20,21]. In this context, bladder ultrasound could serve as an effective, non-invasive, simple, and highly reproducible tool for diagnosing NE, as well as for assessing the outcomes in children and evaluating the rate of therapy response or relapse.

The aim of this review was to assess the role of bladder ultrasound in NE by examining the current evidence in the medical literature, with a particular emphasis on its predictive value regarding therapeutic response and outcomes.

## 2. Materials and Methods

### 2.1. Data Sources

A literature review was conducted to assess the role of ultrasound in children with NE, utilizing five distinct search queries on PubMed with the following keywords: “enuresis & ultrasound”, “enuresis & bladder ultrasound”, “enuresis & bladder wall”, “enuresis & bladder capacity”, and “enuresis & bladder thickness”. To be considered eligible for inclusion, studies had to meet the following criteria: (1) children diagnosed with NE and (2) children who underwent ultrasound evaluation. Exclusion criteria were (1) non-English language publications; (2) children with other urological comorbidities; and (3) studies where ultrasound parameters were not assessed or specified. Abstracts of the identified papers were reviewed by two researchers (IC and PF), who applied the inclusion/exclusion criteria rigorously to determine eligibility for full review. Papers meeting the eligibility criteria were further analyzed in full text by the authors, with any discrepancies resolved through open discussion.

### 2.2. Study Selection

**Figure 1:**
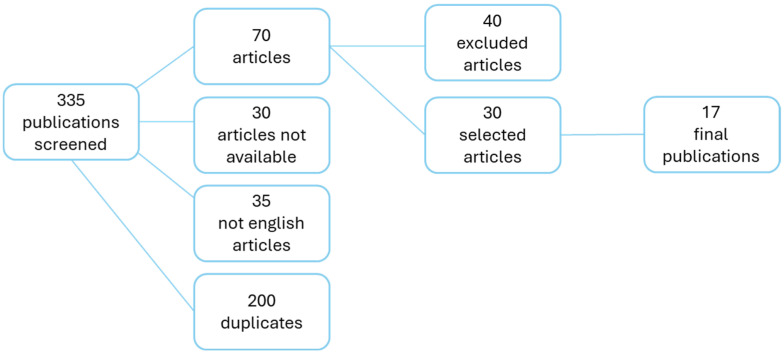
Description of the selection process recruiting publications in our database.

In total, 335 records were identified through database searches. Initially, we excluded 35 articles written in languages other than English, 30 records for which related articles were unavailable, and 200 duplicate papers. In the second step, 40 records were discarded based on the evaluation of titles and abstracts, as they did not meet the inclusion criteria specified. After a further discussion on the reliability of the data, 13 studies were excluded from the remaining 30. As a result, 17 articles were ultimately included in this review. The detailed selection of the medical literature is shown in Figure 1. A wide and extensive summary of all results is shown in Table 1.

### 2.3. Data Extraction

Our data extraction template was filled under strict coordination by the researchers (IC and PF). Data extracted from each eligible paper included: study design, study population sample, mean age, sex prevalence, type of NE and correlation between ultrasound findings and clinical outcome. We also analyzed all study limitations and any eventual conflicts of interest, if reported. In this review, we analyzed the current medical literature on the predictive value of a bladder ultrasound in the response of therapy in enuretic children. A formal ethical approval was not required for our study.

## 3. Results

Out of the seventeen articles included in this review [15,16,20,22,23,24,25,26,27,28,29,30,31,32,33,34,35], ten were clinical studies [16,20,23,24,26,27,28,31,33,34], four were comparative studies [15,22,25,30], two were randomized controlled trials [29,35], and one was a retrospective study [32]. The year of publication varied from 1993 to 2023, with most articles published in the last 20 years (*n* = 13), highlighting the importance of our topic in light of recent scientific evidence. Overall, data from a total of 3100 children were reported, with most studies including a large number of subjects. The total sample included 1414 males and 833 females. One study did not report the sex distribution [15]. The patient population was represented by subjects with non-specified NE [16,22,26,28,32,33], with MNE [15,20,23,24,27,29,30,31,34,35], and NMNE [25]. The therapies used in the studies are heterogeneous and include desmopressin [15,20,23,24,28,34], oxybutynin [22,25,27,32], desmopressin + oxybutynin [29,31,33,35], behavioral therapy [30,32], enuresis alarm [25], and imipramine hydrochloride [26]. One study did not specify the type of therapy used [16]. The main ultrasound criteria analyzed in the studies were: bladder wall thickness index (BVWI), bladder wall thickness (BWT), maximum bladder capacity (MBC), and functional bladder capacity (FBC). Most of the studies have documented an association between ultrasound characteristics of the bladder and the response rate to therapy or relapse, while only two studies have not shown this association [33,34].

## 4. Discussion

NE, commonly referred to as bedwetting, is one of the most prevalent conditions during childhood, affecting approximately 10% of seven-year-olds, with a prevalence dropping to about 0.5 to 2% among teenagers and adults, irrespective of treatment [19,36,37,38,39]. The underlying cause is believed to result from a combination of factors, summarized in three main mechanisms: nocturnal polyuria, where the body produces excess urine during the night; nocturnal detrusor overactivity, where the bladder muscle contracts inappropriately; and a high excitability threshold, which makes it harder for the individual to respond to bladder fullness during sleep [40,41,42,43]. The latter two mechanisms are often linked with reduced bladder capacity, which further complicates the condition. Therefore, the main issue is a mismatch between nighttime urine production and the bladder’s capacity to hold urine, combined with an inability to wake up when the bladder reaches full capacity [38,39,40,41]. These contributing factors to the onset and persistence of NE can all lead to failed treatment outcomes and persistent symptoms [40,41,42,43]. The diagnosis of NE is based on clinical data and the performance of instrumental tests, including UDS. Generally, UDS in pediatric patients is indicated for conditions such as neurogenic bladder-sphincter dysfunction, anorectal malformations, voiding dysfunctions (including urge syndrome and underactive bladder), vesicoureteral reflux, urinary incontinence, infravesical obstruction, or obstructive uropathy [44,45,46]. Recent studies have demonstrated that UDS evaluations are beneficial for enuretic children, particularly those with severe NMNE or therapy-resistant NE, and have recommended UDS for those requiring additional care [44,45,46,47]. In patients with NE, UDS typically reveals storage abnormalities, including reduced bladder capacity, bladder hypocompliance, or detrusor overactivity, which are the principal pathophysiological mechanisms [44,48]. In pediatric patients with NE, the prevalence of low cystometric capacity ranges from 15% to 73%, while detrusor overactivity occurs in 10% to 79% of cases, and low bladder compliance is observed in 2.5% to 40% of patients [18,44,48]. The principal urodynamic tool in NE is uroflowmetry (UFM), a non-invasive test used to measure the rate of urine flow during urination, typically expressed in milliliters per second (mL/s). This test is conducted while the patient is in their usual urination position, reflecting their typical voiding pattern and the volume of urine voided. Post-void residual volume is subsequently measured to assess bladder emptying efficiency [49]. The ICCS recommended standardizing the terminology for abnormal UFM curves, categorizing them as bell (normal), tower, plateau, staccato, and interrupted patterns. However, the classification of UFM curve patterns is still not fully standardized and is primarily based on the physician’s subjective judgment [50]. UFM evaluates several parameters, including the flow pattern, the shape of the flow curve, maximum urinary flow rate (Qmax), volume of urine voided (VV), voiding time (VT), and post-void residual volume (PVR) [49]. While Chang et al. found that an increased PVR is a key predictor of a lower likelihood of achieving a complete response to treatment, regardless of whether the patient has a high Dysfunctional Voiding Symptom Score (DVSS) or not, Elmissiry et al. documented a statistically significant association between daytime symptoms and UDS parameters such as low bladder capacity, detrusor overactivity, and high post-void residual urine [18,33].

Bladder ultrasound is an adjunctive tool to UFM in the diagnostic evaluation and follow-up of NE. Several studies have demonstrated that enuretic children, compared to the control population, have a higher incidence of bladder wall abnormalities and reduced bladder capacity, indicating that these factors are fundamental to the onset of NE. In a comparative study, Acosta et al. found that enuretic patients had a reduced FBC compared to the control group (171.7 mL vs. 225.5 mL; *p* = 0.025). Additionally, it was less than 70% of the expected capacity [38]. Starfield et al. demonstrated a significant difference in bladder capacity between enuretic children and their non-enuretic siblings, as well as between children with primary enuresis and their non-enuretic siblings who do not experience nighttime awakenings [51]. In Kim’s study, it was found that 46.5% of all patients had an FBC below the age-appropriate range, with a higher incidence of reduced FBC in children experiencing daily wetting or frequent nighttime wetting [28]. Kang et al. also observed that 68% to 70% of enuretic children had an age-inappropriate small FBC [52]. Thus, ultrasound has emerged as a novel and promising technique for evaluating bladder dysfunction in enuretic children, with potential implications for treatment management and follow-up [53,54].

### 4.1. Ultrasound Parameters and Multidimensional Indices

The main ultrasound parameters evaluated in enuretic children are FBC, MBC, BWT, and BVWI. In children, bladder capacity grows as they age. Before the age of 8, the bladder capacity increases by around 30 mL each year, starting from a capacity of 30 mL in newborns. The bladder capacity adjusted for age can be estimated using the following formula: PC (proper capacity) = 30 × (age in years)/30 [55]. The MBC refers to the volume of urine voided after consuming the maximum amount of fluid and holding urine for the longest period, reflecting the largest volume voided within hours after fluid intake [56,57,58,59]. The bladder volume is usually determined by evaluating the bladder in both the longitudinal and transverse planes: in the longitudinal plane, the maximum length is measured from the bladder fundus to the internal opening of the urethra, while in the transverse plane, the maximum transverse diameter and the maximum anteroposterior diameter are recorded. Bladder volume index (BVI) is generally determined using the following formula: BVI = Longitudinal section (LS) × Transverse section (TS) × Anteroposterior (AP) [17,34,60]. BWT is typically assessed using a magnified image of the transverse plane of the empty bladder at three locations: anterolateral, lateral, and posterolateral [17,60,61]. On ultrasound, the bladder wall appears as a three-layered structure, with the detrusor muscle visible as a hypoechoic layer situated between two hyperechoic layers that represent the serosa and mucosa [62]. Despite the various potentially applicable approaches, such as measuring the thickness of all three layers or focusing solely on the middle detrusor layer, no significant differences have been found in the thickness of the different bladder wall regions [62]. With low-frequency ultrasound probes (3.5–5 MHz), only significantly thickened bladder walls can be detected. However, high-frequency probes (≥7 MHz) allow for the visualization of even mild bladder wall thickening [63]. Regardless of age or gender, the wall of a normal, empty bladder should measure less than 5 mm, while the wall of a fully distended bladder should measure less than 3 mm [63,64,65,66].

BWT is influenced by bladder filling volume, and therefore, it cannot be used as a standalone diagnostic parameter [17]. Currently, there is no standardized consensus in the literature regarding diagnostic thresholds or reference ranges for BWT based on age and gender [17]. Previous studies suggest that BWT should ideally be measured at a bladder filling volume of 250 mL or more [17,67]. This recommendation is supported by observations in both healthy adults and children, where a decrease in BWT was correlated with an increase in bladder filling volume [17]. In healthy adults, BWT measurements decreased up to 250 mL of bladder filling, while between 250 mL and bladder capacity, BWT remained relatively constant, with statistically insignificant differences [17]. Furthermore, achieving this volume may not always be possible, particularly in patients with detrusor overactivity, leading to potential overestimation or underestimation of the measurement [17,67]. Since image quality depends on ultrasound penetration and frequency, bladder filling volume is expected to affect BWT measurements. As the bladder fills, it ascends from the pelvic region, causing the anterior bladder wall to move closer to the abdominal wall. This increases the likelihood that higher-frequency ultrasound will penetrate more effectively, providing a high-resolution image of the bladder wall. Both this factor and the anatomical thinning of the bladder wall due to distention can impact the accuracy of the measurements [17].

To address these limitations, multiparametric indices have been developed. Kaefer et al. proposed a bladder thickness index, calculated by dividing the average bladder wall thickness—measured at the dome, floor, and both lateral walls—by the internal bladder diameter, based on anteroposterior and transverse dimensions [68]. In contrast, Yeung et al. employed a more complex method to calculate the BVWI [17,69]. Bladder volume at maximum capacity was calculated by multiplying three measurements: sagittal bladder length, maximum transverse diameter, and maximum anteroposterior diameter. The mean BWT was derived by averaging the thicknesses of the anterior, lateral, and posterior walls. BVWI was then obtained by dividing the bladder volume at full capacity by the mean BWT [15,16,17,62]. Thus, BVWI is defined as the ratio of bladder volume at maximum capacity to the mean bladder wall thickness [16,62].

In a study by Yeung et al., this index was validated by classifying 61 children into three groups based on their BVWI. A strong correlation was found between a normal index and normal UDS. Children with a normal BVWI exhibited normal bladder function on UDS, while those with a high BVWI showed bladder overactivity, and children with a low BVWI exhibited a hypocontractile urodynamic pattern [41]. A low index, indicating a small-capacity, thick-walled bladder, was closely associated with a diagnosis of detrusor overactivity on UDS [41]. In another study, among 514 children with primary nocturnal enuresis, 80% with a normal BVWI (ranging from 70 to 130) responded positively to desmopressin. In contrast, 70% of 152 children with abnormal UDS had a BVWI below 70, suggesting a small-capacity, thick-walled bladder [15]. The need to use multiparametric indices instead of a single parameter stems from these observations, which have been corroborated by additional studies. For instance, in a case–control study involving 30 children with PNE and 40 healthy controls, Fahmy et al. found no statistically significant difference in the mean FBC between the pre- and post-treatment groups, and the treatment did not have a significant effect on FBC, while BVWI showed improvement in patients following the administration of both oxybutynin and imipramine, as did the mean BWT [70]. These results are consistent with those of Elsayed et al., who found that FBC, as a single factor, showed no significant correlation with either the severity of enuresis or the response to behavioral therapy. This may be influenced by the fact that bladder volume can vary depending on bladder shape and filling [30]. In contrast, BVWI provided a more accurate correlation with both the severity of enuresis and treatment outcomes [30]. Other studies have further supported the idea that ultrasound-measured bladder volume and bladder wall thickness, when represented by the BVWI, are strongly correlated with the severity of NE and are highly predictive of the response to behavioral therapy [61].

### 4.2. Correlation Between Ultrasound Parameters, Clinical Outcome and Treatment Response

Although it is well-established that enuretic children often exhibit urinary abnormalities, such as reduced functional bladder capacity and increased bladder wall thickness, efforts are underway to correlate the variability of these factors with clinical outcomes. In recent years, several studies have demonstrated the utility of bladder ultrasound, particularly in predicting the response to therapy and the risk of treatment failure, thereby allowing for the potential personalization of intervention strategies.

FBC is one of the most widely researched ultrasound parameters. In a pioneering study by Persson et al. in 1993, it was shown that treatment benefits were strongly associated with ultrasound findings, with bladder capacity having the strongest correlation. The most frequent abnormal finding was a reduced MBC, which was observed in 81% of the children, with 32% showing values below 50% of the expected norm [22]. Similarly, Hamano et al. reported that responders had an FBC of 82 ± 22%, while non-responders had a significantly lower FBC of 56 ± 20% [24]. A further analysis based on volume reduction revealed that 84% of children with a mild reduction in bladder capacity responded well to treatment, compared to only 65% of those with a more significant reduction [22]. also found FBC to be a reliable predictor for desmopressin treatment response, with children having larger bladder capacities more likely to achieve positive results [29]. In a study by Tafuro et al., 65.3% of children were classified as full responders (FR), 25.8% as partial responders (PR), and 8.9% as non-responders (NR). Re-evaluation showed that the FR group had returned to normal ultrasound measurements, while the PR group showed improvements, although their values were still above normal limits. Non-responders exhibited no significant changes [27]. BWT and BVWI are also closely associated with treatment outcomes and clinical severity. Riahinezhad et al. found that increased BWT correlated significantly with detrusor activity and was a strong predictor of a positive response to anticholinergic treatment. In their study, 81.3% of children who responded completely and 76% with good responses had a normal BVWI, while 76.9% of non-responders had a low BVWI. Furthermore, normal BVWI was more commonly observed in children with mild NE, while low BVWI was prevalent in severe cases [20]. Elsayed et al. corroborated these findings, demonstrating that thicker bladder walls were linked to more severe conditions. Among the children with normal BVWI, 97% had a good or complete response, while only 82% of those with low BVWI and 75% of those with high BVWI showed partial or no response [30]. Fuyama et al. further explored the relationship between ultrasound parameters and the healing period, noting that healing time was shorter in children with a BWT of ≥5 mm, indicating a more efficient treatment response [32]. Ultrasound imaging can also inform treatment strategies. Montaldo et al. suggested that a BVWI below 70 indicates small bladder capacity with a thickened bladder wall, implying that combined therapy could effectively manage uninhibited detrusor contractions. This highlights the potential need for anticholinergic medications in children with NE who present reduced bladder capacity and thickened walls [29]. Cayan et al. found that when both bladder capacity and wall thickness increased, children had the most favorable response to treatment with anticholinergic drugs like oxybutynin [71]. Shim et al. showed that the combination therapy group had a higher rate of complete response compared to the monotherapy group after 3 months (44.0% vs. 22.4%, *p* = 0.002), emphasizing the importance of addressing bladder characteristics during treatment. At baseline, there were no significant differences in mean FBC between the two groups. However, the combination therapy group showed a significant increase in bladder capacity, suggesting a better treatment response compared to the monotherapy group [35].

Despite some conflicting data, the majority of studies conducted thus far have demonstrated that the characteristics observed through bladder ultrasound are crucial in the assessment of enuretic children. These ultrasound findings play a significant role in guiding therapeutic decisions and are closely linked to the treatment response rate. Specifically, parameters such as FBC, BWT, and BVWI are frequently used to assess bladder dysfunction and predict outcomes. Each of these parameters provides distinct yet complementary information about the bladder’s structure and function. FBC is a measure of the bladder’s capacity, indicating the volume it can hold before the sensation of fullness leads to the need to void. It is a simple and widely used parameter, often correlated with treatment outcomes such as those seen in desmopressin therapy. A larger bladder capacity typically correlates with better responses to treatment. However, FBC alone fails to address the structural properties of the bladder, such as wall thickness, which can also play a significant role in bladder function. BWT, on the other hand, focuses on the thickness of the bladder wall. It is a critical indicator of bladder health, as increased thickness may suggest bladder dysfunction, such as detrusor overactivity or an abnormal response to filling. BVWI offers a more comprehensive view by combining both bladder volume and bladder wall thickness. This index provides a better understanding of the bladder’s overall function and structure, as it takes into account how the bladder’s capacity and wall thickness interact. Studies have shown that BVWI is strongly correlated with both the severity of NE and treatment response. A low BVWI, indicating a small bladder with thick walls, is often associated with poor treatment outcomes, while a normal or high BVWI tends to correlate with better responses to therapy.

In conclusion, while FBC gives insight into bladder capacity, and BWT provides information on bladder wall health, BVWI integrates both of these factors to give a more comprehensive picture of bladder function. Combining these parameters allows for a more accurate diagnosis and better prediction of treatment outcomes in children with nocturnal enuresis, enabling more tailored therapeutic strategies.

## 5. Limitations

Although our review provides an overview of the most recent evidence regarding the predictive value of bladder ultrasound in children with NE, it has several limitations. First, the screening process may not be exhaustive, and pediatric studies that are not indexed in PubMed or not captured by our keyword searches could have been missed. Therefore, future large-scale studies are needed to further investigate this relationship. Additionally, we did not conduct a meta-analysis due to the heterogeneity of the studies included, especially with respect to sample size, children’s age, type of enuresis, and treatment methods. Furthermore, since ultrasound is operator-dependent, this could introduce a potential bias that must be taken into account.

## 6. Conclusions

Bladder ultrasound has proven to be an important tool in evaluating bladder function and structure in children with NE, providing key information on bladder capacity, wall thickness, and BVWI. These ultrasound parameters correlate with NE severity and treatment outcomes, making them useful in guiding therapeutic decisions. Children with smaller bladder capacities and thicker bladder walls are more likely to benefit from treatments such as anticholinergics, highlighting the potential for personalized treatment approaches. While numerous studies have examined the relationship between ultrasound findings and treatment response, further research is needed to standardize techniques and validate these associations. Future studies should explore the role of bladder imaging in predicting both immediate and long-term treatment success. Current evidence supports the integration of bladder ultrasound into NE management, offering a means to tailor treatments to each child’s specific needs and improve the overall outcomes.

## Figures and Tables

**Table 1:** Studies related to bladder ultrasound in enuretic children.

Study	Design	Sample Size (N)	Mean Age at Diagnosis (Years)	M:F	Patient Population	Ultrasound Measure and Clinical Outcome
Persson-Jünemann et al. (1993) [22]	Comparative study	63	8.5	37:26	NE	The highest response rate is observed in children with a bladder capacity reduction of less than 50%.
Eller et al. (1998) [23]	Clinical study	51	11	37:14	MNE	A strong association was found between a high maximum daytime FBC and a positive response to desmopressin (*p* = 0.006). Among the 11 children with an FBC below 70% of the age-appropriate norm, 7 did not show a response to desmopressin.
Hamano et al. (2000) [24]	Clinical study	114	9.2 ± 2.2	88:26	MNE	At baseline, FBC in responders was 82 ± 22% of the predicted bladder capacity for their age, while in non-responders, it was 56 ± 20% of the expected value for their age.
Yeung et al. (2004) [15]	Comparative study	853	11.2	Not reported	MPNE	A positive response to desmopressin was associated with a BVWI ranging from 70 to 130. In the partial response group, 77% had a BVWI below 70, while in the no response group, 69% had a BVWI below 70.
Vermandel et al. (2005) [25]	Clinical trial	34	9.5	22:12	MPNE	Complete response: an increase in MBC. Partial response: a decrease in MBC. For the 9 children whose primary treatment was ineffective, there was no significant change in MBC. The MBC values before and after primary treatment were 339 (32) mL and 294 (112) mL, respectively, resulting in a reduction in normalized capacity from 110 (31)% to 67 (26)%.
Miyazato et al. (2007) [26]	Clinical study	77	9.9	53:24	PNE	Children with ultrasonographic bladder abnormalities showed a poorer response to treatment (*p* = 0.005). Those with bladder wall thickening were less likely to respond to treatment (*p* = 0.035).
Sreedhar et al. (2008) [16]	Clinical study	35	9.03	23:12	PNE	All children with a normal BVWI had either a complete or good response to treatment, whereas 62.5% of those with an index below 70 did not respond to the treatment.
Tafuro et al. (2010) [27]	Comparative study	455	9.58	282:173	NMPNE	75.7% of full responders had normal bladder wall thickness. 18.1% of partial responders showed no improvement in bladder wall thickness. 6.2% of nonresponders had a consistently thickened bladder wall.
Kim et al. (2012) [28]	Clinical study	101	7.7 ± 2.3	72:29	NE	The FBC decreased in relation to the severity of NE (*p* = 0.007).
Montaldo et al. (2012) [29]	Randomized controlled trial	206	10.6 ± 2.9	117:89	MNE	The BVWI and average bladder capacity for age were predictors of a stronger response to the combination of oxybutynin and desmopressin.
Elsayed et al. (2012) [30]	Comparative study	122	Not reported	68:54	MNE	Among those with a complete response 81.3%, and with a good response 80% had a normal BVWI. In contrast, 80% of those who did not respond to treatment had a low BVWI. Out of 34 children with a normal BVWI, 33 (97%) had a complete or good response, while 23 of 28 children with a low BVWI (82%) and 9 of 12 children with a high BVWI (75%) experienced a partial or no response to treatment.
Kovacevic et al. (2014) [31]	Clinical study	279	9.56 ± 2.69	178:101	MPNE	Children with RBUS abnormalities seem to be less responsive to treatment compared to enuretic children with normal RBUS (*p* = 0.002).
Fuyama et al. (2018) [32]	Retrospective study	117	6.6 ± 1.43	76:41	NE	The recovery period was notably shorter in the group with bladder wall thickness greater than 5 mm compared to those with bladder wall thickness less than 5 mm.
Chang et al. (2018) [33]	Clinical study	100	8.5 ± 2.3	66:34	PNE	There was no notable correlation between a reduced FBC and the response to medical treatment.
Liu et al. (2021) [34]	Clinical study	322	8.29 ± 2.37	191:131	MNE	FBC did not serve as a predictor for the response to desmopressin.
Shim et al. (2021) [35]	Randomized Controlled Trial	99	7.51 ± 1.83	65:34	MPNE	An increase in FBC (30% or more, 6 months after stopping treatment compared to baseline) was linked to a lower likelihood of relapse.
Riahinezhad et al. (2023) [20]	Clinical study	72	6.37 ± 2.7	39:33	MNE	Among children with complete responses, 81.3% had a normal BVWI, and 76% of those with good responses also had a normal BVWI. In contrast, 76.9% of children who did not respond to treatment had a low BVWI.

Nocturnal enuresis (NE), MNE (monosymptomatic nocturnal enuresis), PNE (primary nocturnal enuresis), monosymptomatic primary nocturnal enuresis (MPNE), wall thickness index (BVWI), maximum bladder capacity (MBC), non-monosymptomatic primary nocturnal enuresis (NMPNE), renal/bladder ultrasound (RBUS), functional bladder capacity (FBC).

## Data Availability

Not applicable.
